# Broadband optical frequency comb generation based on single electro-absorption modulation driven by radio frequency coupled signals

**DOI:** 10.1007/s12200-022-00045-0

**Published:** 2022-11-14

**Authors:** Pan Jiang, Peili Li, Yiming Fan

**Affiliations:** College of Electronic and Optical Engineering & College of Microelectronics, Nanjing University of Posts and Telecommunications, Nanjing, 210023 China

**Keywords:** Optical frequency comb (OFC), Electro-absorption modulator (EAM), Radio frequency (RF) coupled signal

## Abstract

Broadband optical frequency comb (OFC) generation based on a single electro-absorption modulator (EAM) is proposed. The EAM is driven by a radio frequency (RF) multi-frequency signal generated by a multiplication coupler composed of an electrical power splitter and an arithmetic circuit. Thus the number of comb-lines of the generated OFC can be increased. A complete theoretical model of OFC generation by an EAM driven by *n*th power of the RF source is established, and the performance of the OFC is analyzed by using OptiSystem software. The results show that, the number of comb-lines of the OFC is positively correlated with the number of multiplication of the RF source signal. The frequency spacing of the comb-lines is twice the frequency of the RF source signal and is tunable by adjusting the frequency of the RF source signal. Increasing chirp factor and modulation index of EAM could increase the number of comb-lines of the generated OFC. The amplitude of the RF source signal had little impact on the flatness of the OFC and the average OFC power. The scheme developed is not only simple and low-cost, but also can produce a large number of comb-lines.

**Graphical Abstract:**

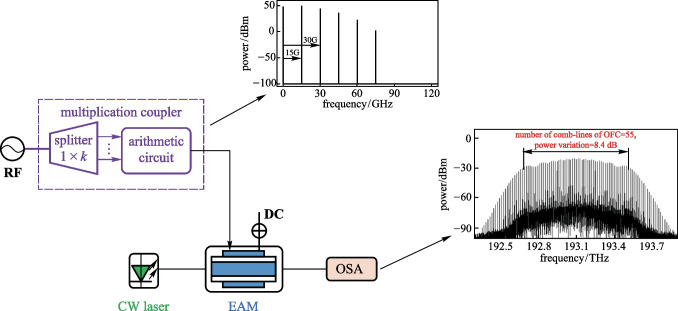

## Introduction

Optical frequency comb (OFC) is a spectrum composed of a series of evenly spaced discrete frequency components with coherent and stable phase relationship, which is widely used in optical communication, optical arbitrary waveform generation, microwave photon signal processing and high precision optical measurement [1–5].

High-quality OFC with a large number of comb lines, high flatness of comb line power, and tunable frequency spacing is the current research focus. OFC can be generated based on mode-locked laser [6, 7], nonlinear fiber [8], micro-ring resonator [9], photoelectric oscillator [10, 11], and external modulator [12–19]. Among these methods, using external modulator has the advantages of stable output spectrum, adjustable frequency spacing between comb lines, simple structure, and flexible operation.

In Ref. [12], an OFC with 5 comb-lines and flatness of 0.6 dB was generated based on a dual parallel Mach–Zehnder modulator (DPMZM) driven by a single RF source [12]. And in Ref. [13], an OFC was generated by a polarization modulator (PolM) which was also driven by a single RF source. 7 comb-lines with flatness of 1.78 dB was obtained [13]. Generally, the number of comb-lines obtained by using external modulator with a single RF source signal is small. To obtain OFC with more comb-lines, it was proposed to use multiple RF source signals to drive a single modulator jointly, or use cascaded modulators. In Ref. [14], an OFC with 9 comb-lines and flatness of 1.1 dB was produced based on a dual-drive Mach–Zehnder modulator (DDMZM) driven by two RF source signals with different frequencies [14]. Similarly, in Ref. [15], by using two RF source signals to drive a phase modulator (PM), an OFC with 11 comb-lines and flatness of 1.9 dB was realized [15]. In Ref. [16], by cascading a nonzero chirp electro-absorption modulator (EAM) and a PM driven by a single RF source signal, an OFC with 21 comb-lines and flatness of 5 dB was obtained [16]. And in Ref. [17], a nonzero chirp EAM and two PMs driven by a single RF source signal was used to produce an OFC with 21 comb-lines and flatness of 0.18 dB [17]. Although the use of multiple RF source signals or cascaded modulators can increase the number of comb-lines, the overall cost also increases.

In this paper, OFC generation based on a nonzero-chirp EAM driven by a RF coupled signal was proposed. Low-cost electrical power splitter and arithmetic circuit was cascade, which we call a multiplication coupler, and was used to generate a multi-frequency signal. The multi-frequency signal generated by the multiplication coupler was called RF coupled signal, and was used to drive the EAM to increase the number of comb-lines. In contrast to lithium-niobate (LiNbO_3_) MZM, EAM has the advantages of low cost and integrability [11]. In previous studies, nonzero-chirp EAM was adopted [16, 17]. While in this scheme, an EAM with positive-chirp was adapted. A theoretical model was established to investigate OFC generation based on positively chirped EAM driven by the RF coupled signal. The effects of frequency and amplitude of the RF source signal, the number of multiplications of the RF source signal, the chirp factor and modulation index of the EAM on the number of comb-lines and the spectral bandwidth of the OFC were investigated by using OptiSystem software.

## Operating principle

**Fig. 1:**
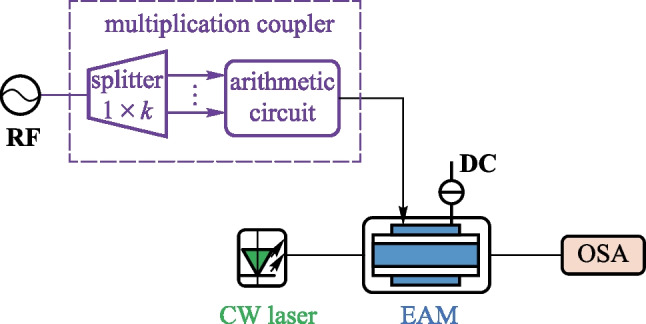
Schematic diagram of the OFC generation scheme based on a single EAM and a multiplication coupler composed of an electrical power splitter and an arithmetic circuit. *RF* radio frequency source; *splitter* electrical power splitter; *CW laser* continuous wave laser; *EAM* electro-absorption modulator; *DC* direct current voltage source; *OSA* optical spectrum analyzer

Figure 1 shows schematic diagram of the OFC generation scheme proposed in this work. The sinusoidal electrical signal outputted from a RF source enters the multiplication coupler. It is divided into *k* branches with equal power by an electrical power splitter. Then the *n*th power of the RF source signal (i.e., the RF coupled signal) is generated by an arithmetic circuit consisting of a squaring circuit and an electrical multiplier. Since OFC generated using this scheme is not ideal when *n* is odd, we only study the case when *n* is even and *n* ≥ 2. The optical field from a continuous wave (CW) laser enters an EAM, where the intensity and phase of the optical field are modulated by the RF coupled signal. New frequency components are then generated in the output optical signal of the EAM. By adjusting *n*, as well as the chirp factor and modulation index of the EAM, the number of comb-lines of the OFC can be changed, and the power of adjacent sidemodes can be uniform. A tunable OFC with a large number of comb-lines and wide spectrum bandwidth can be generated.

Mathematically, the sinusoidal electrical signal output from the RF source is expressed as

1 $$V_{{{\text{RF}}}} \left( t \right) = V_{0} \sin \left( {2\uppi f_{{\text{c}}} t} \right),$$

where *V*_0_ and *f*_c_ are the amplitude and frequency of the RF source signal, respectively.

The RF coupled signal generated is the *n*th power of the RF source signal, and can be expressed as

2 $$V_{{{\text{multi}}}} \left( t \right) = V_{{{\text{EAM}}}} \sin^{n} \left( {2{\uppi }f_{{\text{c}}} t} \right),$$

where *V*_EAM_ represents the amplitude of the RF coupled signal (i.e., the modulation voltage of EAM), $n \ge 2$ and *n* is an even number. The value of the *V*_EAM_ is related to *V*_0_ and the detailed configuration of the arithmetic circuit.

The input CW input optical field is modulated in EAM, and the output optical field can be expressed as

3 $$E_{{{\text{out}}}} (t) =\; E_{{{\text{in}}}} (t)\sqrt {(1 - m) + mV_{{\bmod }} (t)} \exp \left\{ {{\text{j}}\frac{\alpha }{2}\ln [(1 - m) + mV_{{\bmod }} (t)]} \right\} ,$$

where *E*_in_(*t*) is the input optical field from the CW laser source denoted as $E_{{{\text{in}}}} \left( t \right) = E_{0} \exp \left( {\text{j}2\uppi f_{0} t} \right)$, *E*_0_ and *f*_0_ are the amplitude and center frequency of the optical field, respectively. *V*_mod_(*t*) represents the voltage applied to the EAM, which is given by $V_{\bmod } \left( t \right) = V_{{{\text{bias}}}} + V_{{{\text{multi}}}} \left( t \right)$, where *V*_bias_ represents the DC bias voltage of the EAM. *m* is the modulation index of EAM denoted as $m = {{V_{{{\text{EAM}}}} } \mathord{\left/ {\vphantom {{V_{{{\text{EAM}}}} } {V_{{{\text{bias}}}} }}} \right. \kern-\nulldelimiterspace} {V_{{{\text{bias}}}} }}\left( {0 < m < 1} \right)$. $\alpha$ is the chirp factor of EAM. When the chirp factor of EAM is close to 0, the complex exponential part of Eq. (3) approaches 1, and only intensity modulation occurs in the EAM, and new frequency components cannot be generated [16]. When the chirp factor of EAM is nonzero, not only intensity modulation but also phase modulation occurs in the EAM. Thus, with the increase of the chirp factor, the number of frequency components in the output spectrum increases [11, 16–19].

Equation (3) reveals that the modulation index of EAM can affect the amplitude and phase of the output optical field. To simplify the theoretical model, *m* is set close to 1, such that the DC bias voltage is approximately equal to the modulation voltage of the EAM. We can define $f\left( {V_{\bmod } \left( t \right)} \right) = \ln \left[ {V_{\bmod } \left( t \right)} \right]$, and Eq. (3) can be written as

4 $$E_{{{\text{out}}}} \left( t \right) = E_{{{\text{in}}}} \left( t \right)\sqrt {V_{\bmod } \left( t \right)} \exp \left[ {{\text{j}}\frac{\alpha }{2}f\left( {V_{\bmod } \left( t \right)} \right)} \right].$$

The Taylor series expansion of *f*(*V*_mod_(*t*)) at $V_{\bmod } \left( t \right) = V_{{{\text{bias}}}}$ can be expressed as

5 $$f\left( {V_{\bmod } \left( t \right)} \right) = \sum\limits_{{\delta { = }0}}^{ + \infty } {\left( {\delta !} \right)^{ - 1} f^{\left( \delta \right)} \left( {V_{{{\text{bias}}}} } \right)\left[ {V_{{{\text{EAM}}}} \sin^{n} \left( {2{\uppi }f_{{\text{c}}} t} \right)} \right]^{{^{\delta } }} ,}$$

where *f*^(*δ*)^(*V*_bias_) is the *δ*th order derivative of *f*(*V*_mod_(*t*)) at $V_{\bmod } \left( t \right) = V_{{{\text{bias}}}}$. In Eq. (5), we get

6 $$\begin{aligned}&f^{\left( \delta \right)} \left( {V_{{{\text{bias}}}} } \right) \cdot V_{{{\text{EAM}}}}^{\delta } \\&\quad= \left\{ \begin{gathered} \ln \left( {V_{{{\text{bias}}}} } \right),\delta = {0}, \hfill \\ - \left( {\delta - 1} \right)!\left[ {\frac{{\sin^{n} \left( {2{\uppi }f_{{\text{c}}} t} \right)}}{m} + \sin^{n} \left( {2{\uppi }f_{{\text{c}}} t} \right)} \right]^{ - \delta } ,\,\delta = 2,4, \ldots, \hfill \\ \left( {\delta - 1} \right)!\left[ {\frac{{\sin^{n} \left( {2{\uppi }f_{{\text{c}}} t} \right)}}{m} + \sin^{n} \left( {2{\uppi }f_{{\text{c}}} t} \right)} \right]^{ - \delta } ,\,\delta = {1},3,5, \ldots. \hfill \\ \end{gathered} \right.\end{aligned}$$

It can be seen from Eq. (6) that the magnitude of *f* ^(*δ*)^(*V*_bias_)·*V*_EAM_^*δ*^ is independent of the value of *V*_EAM_. That is, *f*(*V*_mod_(*t*)) does not vary with the change of *V*_EAM_.

According to the Power-Reducing formula for trigonometric functions, the sin^*nδ*^(2π*f*_c_*t*) in Eq. (5) can be expanded to give

7 $$\begin{aligned} \sin^{n\delta } \left( {2{\uppi }f_{{\text{c}}} t} \right) = \, & C_{0} + C_{2} \sin \left( {2 \cdot 2{\uppi }f_{{\text{c}}} t} \right) \\ & + \ldots + C_{n\delta } \sin \left( {n\delta \cdot 2{\uppi }f_{{\text{c}}} t} \right),\,\delta = 0,1,2, \ldots , + \,\infty . \\ \end{aligned}$$

According to the Jacobi-Angel identity, the output light field of EAM can be represented by Bessel functions:

8 $$\begin{aligned} E_{\text{out}} (t) =\; & E_{\text{in}} (t)\sigma (t)\exp \left\{ {\text{j}\frac{\alpha }{2}\left[ {\sum\limits_{{k = 1}}^{{ + \infty }} {A_{k} \sin (4\uppi kf_\text{c} t)} } \right]} \right\} \\ =\; & E_{\text{in}} (t)\sigma (t)\sum\limits_{{u_{k} = - \infty ,k = 1}}^{{ + \infty }} {J_{{u_{1} }} (\psi _{1} )J_{{u_{2} }} (\psi _{2} )} ...J_{{u_{k} }} (\psi _{k} )\\&\times\exp \left[ {\text{j}4\uppi (u_{1} + u_{2} + ... + u_{k} )f_\text{c} t} \right], \\ \end{aligned}$$

where *σ*(*t*) is the variation of amplitude denoted as $\sigma (t) = \sqrt {V_{\bmod } (t)} \exp \left( {{\text{j}}\frac{\alpha }{2}A_{0} } \right)$. *u*_*k*_ and $\psi_{k} = {{\alpha A_{k} } \mathord{\left/ {\vphantom {{\alpha A_{k} } 2}} \right. \kern-\nulldelimiterspace} 2}$ are the order and argument of the Bessel function of the first kind, respectively, where *A*_*k*_ represents the constant coefficient of the polynomials in Eq. (5). Combined with the Power-Reducing formula for the trigonometric function, it can be seen from Eq. (8) that $\sqrt {V_{\bmod } (t)}$ in *σ*(*t*) will only vary in a small range with the change of *V*_EAM_. Expending Eq. (2) using the Power-Reducing formula, it can be found that $\sqrt {V_{\bmod } (t)}$ in *σ*(*t*) only vary in a small range with the change of *V*_EAM_. As the amplitude of the Bessel function of first kind is limited between about − 0.4 and 1, *V*_EAM_ has a minimal effect on the power of the each optical sidemode and flatness of the OFC.

Equation (8) reveals that the generated OFC is the superposition of *k* spectra with frequency spacings of 2*f*_c_, 4*f*_c_, 6*f*_c_, …, (2*k* − 2)*f*_c_ and 2*kf*_c_. Obviously, the frequency spacing between comb-lines of the overall OFC is determined by the minimum frequency spacing of 2*f*_c_. When the modulation index of the EAM approaches 1, and the EAM has large chirp factor, OFC with large number of comb-lines, wide spectrum coverage, and tunable frequency spacing can be obtained.

## Simulation results and discussion

The proposed broadband OFC generation scheme based on single EAM driven by a RF coupled signal was simulated by using the OptiSystem software. In the simulation, the center frequency of CW Laser was 193.1 THz, the linewidth was 10 MHz, and the optical power was 0 dBm. The RF source output was a sinusoidal electrical signal with amplitude of 2 V and frequency of 7.5 GHz, and a multiplication coupler was used to generate 10th power of the RF source signal. The modulation index and chirp factor of EAM were 0.99 and 16, respectively. The resolution of optical spectrum analyzer (OSA) was 10 MHz. All parameters of the components in the simulation remained unchanged unless otherwise stated. Note that the number of comb-lines of OFC in the all simulation results is calculated based on the criterion that the optical power of the highest-order comb line should be no less than the minimum optical power of the comb line between the highest- and the 0th order comb line.

### Effects of the number of coupling of the RF coupled signal

**Fig. 2:**
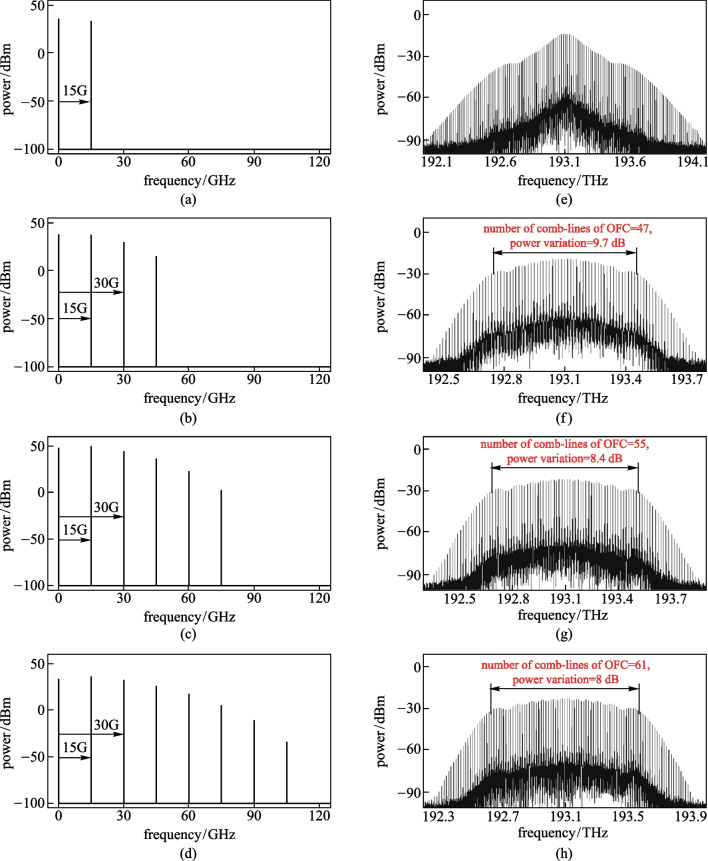
Influence of varying the number of multiplication times of the RF source signal on the generated OFC. **a**–**d** The frequency spectrum for 2nd, 6th, 10th, 14th power of the RF source signal, respectively. **e**–**h** The corresponding optical spectra generated by using the *n*th power of the RF source signal as the modulation signal of the EAM

Figure 2 shows the power spectrum of *n*th power of the RF coupled signal and the output optical spectrum of generated OFC, for *n* = 2, 6, 10, and 14. Figures 2a–d suggest that the frequency components in the RF coupled signal increases with *n*, as the number of multiplication operations increased. The frequency spacing between adjacent frequency components was 15 GHz, which was twice the frequency of the RF source signal. As shown in Figs. 2c–h, the frequency spacing between the spectral lines in the generated optical spectrum was equal to the frequency spacing of the adjacent RF frequency components, i.e., 15 GHz, which equals twice the frequency of the RF source. Therefore, the bandwidth of the spectral lines could be broadened by increasing the frequency components of the RF coupled signal. When the EAM was driven by the 2nd power of the RF source signal, the envelope of the generated optical spectrum was not flat at the top, so OFC was not formed, as shown in Fig. 2e. With the increase of the *n*, the number of comb-lines of OFC was 47, 55, and 61, and the corresponding flatness of the OFC were 9.7, 8.4, and 8 dB, respectively, as shown in Figs. 2f–h. The main reason was that with increase of n, the frequency components of the RF coupled signal also increased. This affected the optical power of each comb line. That is, the optical power of low-order comb lines decreased, and the optical power of high-order comb lines increased. The number of comb-lines of OFC and the spectral bandwidth of the OFC could be increased by increasing the number of multiplication times of RF source signal. The RF coupled signal generated by the multiplication coupler can be equivalent to a multi-frequency RF signal. However, the squaring circuit and the multiplier used in the arithmetic circuit can be realized by commercialized products, and the cost is lower than using multiple RF sources.

### Effects of the output frequency of the RF source signal

**Fig. 3:**
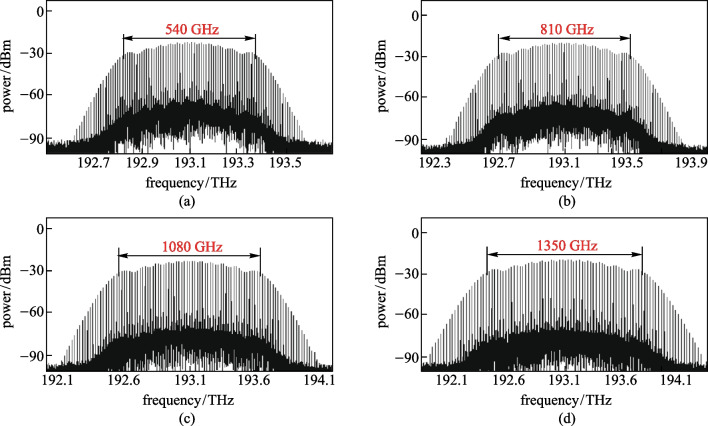
Influence of the frequencies of the RF source signal on the optical spectrum of the OFC. **a** 5 GHz, **b** 7.5 GHz, **c** 10 GHz, **d** 12.5 GHz

Figures 3a–d show the optical spectrum generated when the frequency of the RF source is 5, 7.5, 10, and 12.5 GHz, respectively. Table 1 lists the frequency spacing, number of comb-lines, flatness and spectral bandwidth of the generated OFC for different frequencies of the RF source signal. As the frequency of the RF source signal increased, OFC with 55 comb-lines could be generated, and the corresponding flatness of the OFC were 8.39, 8.4, 8.39, and 8.38 dB, respectively. The frequency spacing of the OFC increased as the frequency of the RF source signal increased, with values of 10, 15, 20, and 25 GHz respectively. The spectral bandwidth of OFC widened as the output frequency of the RF source signal increased, with values of 540, 810, 1080, and 1350 GHz, respectively. The results show that tunable OFC could be achieved by changing the frequency of the RF source signal.

**Table 1:** Comparison of the frequency spacing, number of comb-lines, flatness and spectral bandwidth of the OFC for different frequencies of the RF source signal

RF source signal frequency/GHz	Frequency spacing/GHz	Number of comb-lines	Flatness/dB	Spectral bandwidth/GHz
5	10	55	8.39	540
7.5	15	55	8.4	810
10	20	55	8.39	1080
12.5	25	55	8.38	1350

### Effects of the chirp factor of EAM

**Fig. 4:**
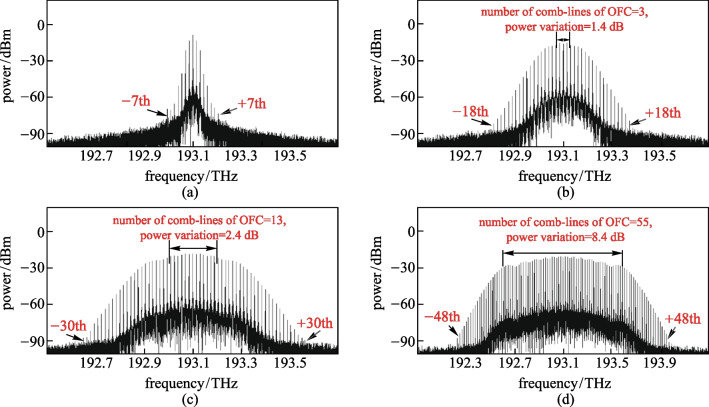
Influence of the chirp factor of EAM on the optical spectrum of the OFC. Chirp factor of **a** 0, **b** 4, **c** 8, **d** 16 are considered

Figures 4a–d show the optical spectrum generated when the chirp factor of EAM was 0, 4, 8 and 16, respectively. When the chirp factor of EAM was 0, the highest sidemode in the optical spectrum that could be observed was ± 7th-order, and no OFC was formed, as shown in Fig. 4a. With increase of the chirp factor, the power of higher-order optical sidemode increased and the power of lower-order optical sidemode decreased; the number of sidemodes in the optical spectrum and the number of comb-lines of OFC increased. As can be seen from Fig. 4d, when the chirp factor of EAM increased to 16, the ± 48th-order optical sidemode could be observed at most, and the number of comb-lines and flatness of OFC were 55 and 8.4 dB, respectively. This was due to phase-modulation of the input light in a nonzero-chirped EAM, in addition to intensity modulation, which affected the power distribution over different optical sidemodes. By adopting EAM with a high chirp factor, OFC with a large number of comb-lines and wide bandwidth could be obtained.

### Effects of the modulation index of EAM

**Fig. 5:**
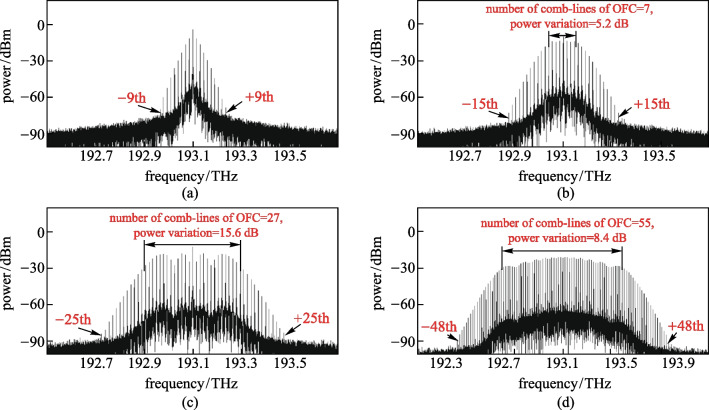
Influence of the modulation index of EAM on the optical spectrum of the OFC. Modulation index of **a** 0.2, **b** 0.5, **c** 0.8, **d** 0.99 are considered

Figures 5a–d show the optical spectra of generated OFC when the modulation indices of EAM were 0.2, 0.5, 0.8, and 0.99 respectively. In the case that the modulation voltage remained unchanged, the modulation index could be adjusted by changing the DC bias voltage of the EAM. When the modulation index of EAM was 0.2, the ± 9th-order optical sidemode could be observed at most, and the envelope of the spectrum was not flat at the top, as shown in Fig. 5a. With increase of the EAM modulation index, the number of sidemodes in the optical spectrum and the number of comb-lines of OFC increased. As can be seen from Fig. 5d, when the modulation index of EAM increased to 0.99, the ± 48th-order optical sidemodes could be observed at most, and an OFC with 55 comb-lines and flatness of 8.4 dB was generated. By decreasing the DC bias voltage of the EAM, the modulation index increased, and the number of comb-lines and the spectral bandwidth of OFC could be increased.

### Effects of the amplitude of RF source signal

**Fig. 6:**
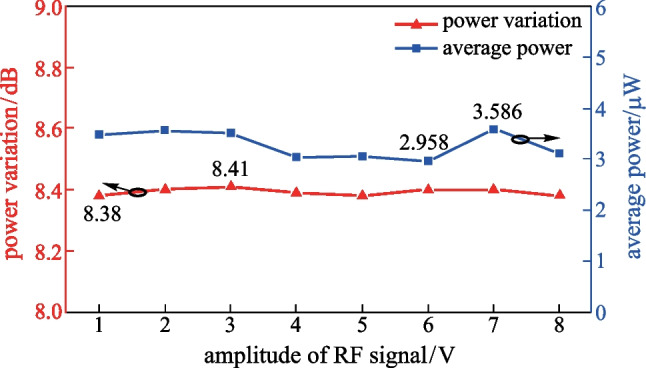
Influence of the amplitude of the RF source signal on the flatness and average power of OFC

When the amplitude of the RF source signal changed from 1 to 8 V, OFC with 55 comb-lines were always generated. The flatness and average OFC power (average optical power of all comb-lines) are shown in Fig. 6. The DC bias voltage of the EAM should be approximately equal to the modulation voltage of EAM to ensure the modulation index of 0.99. It could be observed that the flatness of the OFC changes within only 0.03 dB and the average OFC power fluctuated between 2.958 and 3.586 μW, as the amplitude of the RF source signal changed. The small variation of flatness and average power of OFC was in agreement with the theoretical analysis and can be attributed to the fact that *V*_EAM_ had minimal impact on the power distribution over different optical sidemodes, while *V*_EAM_ is directly related to the amplitude of RF source signal according to Eqs. (1) and (2). As a result, this scheme can achieve broadband OFC even with low power RF source signal.

## Conclusions

In this paper, an approach for generating broadband OFC based on single EAM driven by a multi-frequrency RF signal is proposed. The theoretical model is established and the effects of the frequency and amplitude of the RF source signal, the number of multiplications of the RF source signal, the chirp factor and modulation index of the EAM on the OFC are explored by using OptiSystem software. The results show that the number of comb-lines of the OFC can be increased by increasing the number of multiplications of the RF source signal. The frequency spacing of the optical comb-line is twice the frequency of the RF source signal. As the chirp factor and modulation index of EAM increase, the number of comb-lines of OFC increase. The change of the amplitude of the RF source signal has little effect on the flatness and average power of the OFC. This scheme can generate OFC with a large number of comb-lines. It also has the advantage of simple structure and low cost due to the use of EAM and the multiplication coupler. This scheme can be applied to broadband and flat OFC generation with low power RF source.
